# Case Report: Bilateral targeted intraoperative radiotherapy: a safe and effective alternative for synchronous bilateral breast cancer

**DOI:** 10.3389/fonc.2023.1276766

**Published:** 2023-10-24

**Authors:** Fardeen Bhimani, Maureen McEvoy, Anjuli Gupta, Jessica Pastoriza, Areej Shihabi, Amar Basavatia, Wolfgang A. Tomé, Jana Fox, Keyur Mehta, Sheldon Feldman

**Affiliations:** ^1^Breast Surgery Division, Department of Surgery, Montefiore Medical Center, Montefiore Einstein Center for Cancer Care, Bronx, NY, United States; ^2^Department of Radiation Oncology, Montefiore Medical Center, Bronx, NY, United States

**Keywords:** breast cancer, intraoperative radiotherapy, bilateral breast cancer, bilateral IORT, TARGIT, IORT, bilateral breast irradiation

## Abstract

**Background:**

The incidence of bilateral breast cancer (BBC) ranges from 1.4% to 11.8%. BBC irradiation is a challenge in current clinical practice due to the large target volume that must be irradiated while minimizing the dose to critical organs. Supine or prone breast techniques can be used, with the latter providing better organ sparing; both, however, result in lengthy treatment times. The use of Intra-operative radiotherapy (IORT) in breast cancer patients who choose breast conservation has been highlighted in previous studies, but there is a scarcity of literature analyzing the utility and applicability of IORT in BBC. This case series aims to highlight the applicability of administering bilateral IORT in patients with BBC.

**Case reports:**

Five patients with bilateral early-stage breast cancer (or DCIS) were treated with breast-conserving surgery followed by bilateral IORT. Of the 10 breast cancers, 8 were diagnosed as either DCIS or IDC, while the other 2 were diagnosed as invasive lobular carcinoma and invasive carcinoma, respectively. During surgery, all patients received bilateral IORT. Furthermore, 1 patient received external beam radiation therapy after her final pathology revealed grade 3 DCIS. The IORT procedure was well tolerated by all five patients, and all patients received aromatase inhibitors as adjuvant therapy. Additionally, none of these patients showed evidence of disease after a 36-month median follow-up.

**Conclusion:**

Our findings demonstrate the successful use of IORT for BCS in patients with BBC. Furthermore, none of the patients in our study experienced any complications, suggesting the feasibility of the use of IORT in BBC. Considering the benefits of improved patient compliance and a reduced number of multiple visits, IORT may serve as an excellent patient-centered alternative for BBC. Future studies are recommended to reinforce the applicability of IORT in patients with BBC.

## Introduction

Breast cancer is the most commonly diagnosed malignancy among women across the United States (1). To date, breast cancer surgery remains the only definitive treatment option. For many years, mastectomy was perceived as the only treatment option, even for early-stage breast cancer. However, after multiple studies documented similar long-term outcomes in terms of locoregional recurrence and survival, a more conservative surgical approach is being used for small breast cancers. This has led to a transition from surgical management with a mastectomy to a lumpectomy for early-stage breast cancer. Breast-conserving surgery (BCS), followed by adjuvant radiotherapy, has been shown to be as effective as mastectomy in terms of oncological outcomes (2–4). This paradigm shift applies not only to conservative surgical approaches but also to patients undergoing radiation therapy after BCS. Although the majority of the patients receive whole-breast irradiation (WBI) following BCS, results from recent prospective clinical trials have led to the increased application of partial breast irradiation (PBI) in selected patients.

Intraoperative radiotherapy (IORT) is another radiation modality for partial breast irradiation that has gained popularity in recent years. The advantage of utilizing IORT is that it allows for a single dose to be administered at the time of lumpectomy. The inherent merits of normal tissue preservation and breast conservation, as well as convenience, have led IORT to become a popular treatment option for patients (5–8). Importantly, a study conducted by the TARGIT group on 2298 patients found that TARGIT-IORT was non-inferior to external beam radiation therapy (EBRT), with a local recurrence rate of 2.11% for TARGIT-IORT vs. 0.95% for EBRT (9).

Patients with primary breast cancer are more likely to develop contralateral primary breast cancers, such as synchronous breast cancer (SBC) or metachronous breast cancer (MBC) (10). The time of diagnosis from primary cancer distinguishes both of these contralateral cancers. SBC is diagnosed within 6 months of the primary diagnosis, while MBC is diagnosed after 6 months (11, 12). The incidence of patients diagnosed with BBC across various studies ranges from 1.4% to 11.8% (13–18). The optimal surgical management in patients with BBC is not well delineated. Patients undergoing BCS for BBC may experience a challenging decision-making process due to factors such as locoregional recurrence in each primary cancer and bilateral breast irradiation. Despite the lack of consensus, it has been proposed that therapy in BBC be similar to the treatment strategy for unilateral breast cancer (19). Furthermore, patients receiving bilateral breast irradiation for BBC vs. unilateral breast cancer have shown similar outcomes (20). However, the role of radiotherapy in patients older than 65 years can be controversial. The PRIME II study conducted on patients older than 65 years demonstrated deleterious effects on local recurrence (9.8% vs. 0.9%) without significantly impacting the overall survival (21). Therefore, a shared decision-making process with patients who are made aware of the risks of standard radiotherapy, as well as its omission, is required.

The use of IORT in BCS has been well demonstrated in previous trials, but there is a paucity of literature highlighting its utility in bilateral breast cancer (BBC). The non-inferior treatment approach of IORT that allows for better critical organ sparing, fewer side-effects and better patient compliance could potentially be a treatment option for patients diagnosed with BBC. Thus, our case series aims to highlight the applicability of administering bilateral IORT in patients with BBC. Patients receiving IORT at our institution are enrolled on a registry trial, with eligibility limited to ER+ clinically node-negative invasive carcinomas up to 3.5cm in maximal dimension as per the TARGIT-A criteria (22), and ER+ DCIS, grades 1-2, measuring up to 2.5cm, as per the ASTRO consensus partial breast irradiation guidelines (23).

## Case reports

### Patient 1

A 58-year-old female presented with right breast calcifications spanning 2cm on mammography. Subsequent MRI identified a 7mm left breast enhancing mass. Biopsy of both breasts demonstrated clinical stage 0 Tis N0 grade 2, ER+, PR+ ductal carcinoma *in situ* (DCIS). Wide local excision was performed on each site, and the Intrabeam 600 system (Zeiss, Oberkochen, Germany) delivered IORT sequentially. A 35-mm spherical applicator delivered 20 Gy to the surgical margin during each IORT treatment. No sentinel lymph node biopsy (SLNB) was performed **Table 1**.

**Table 1:** Summary of patient data.

Patient	Age (years)	Final Pathology For Right/Left Side	Pathological Size (mm) For Right/Left Side	Size of the Tumor Resected (cm) For Right/Left Side	Applicator Size (mm) for Right/Left Side	Additional Radiation with External Beam	Complications	Adjuvant Therapy
1.	58	Ductal Hyperplasia/DCIS	NA^~^/30	4.8 x 4.5 x 11/3.5 x 3.3 x 1.5	35/35	Yes	No	Aromatase Inhibitor + EBRT^+^
2.	72	IDC + DCIS/IDC	6/17	6.1 x 3.7 x 2.3/5 x 4.2 x 2	35/35	No	No	Aromatase Inhibitor
3.	73	IDC/ILC	15 x 8/16	5.5 x 4.3 x 1.5/7 x 3.7 x 0.9	30/35	No	No	Aromatase Inhibitor
4.	69	IDC/IDC	3 x 2/8 x 7 x 6	4.1 x 3.2 x 1.1/4 x 3 x 1	30/35	No	No	Aromatase Inhibitor
5.	77	Invasive carcinoma*/ILC	12/30	5.8 x 3.8 x 2/5.5 x 4.9 x 2.8	40/45	No	No	Aromatase Inhibitor

*Admixed ductal & lobular features.

~ Not Available.

^+^ External Beam Radiation Therapy.

IORT and surgery were uneventful. The histology of the left breast revealed pathological Stage 0 Tis N0 grade 3 DCIS spanning 3 cm with focal necrosis and a rare focus early microinvasion that could not be ruled out. The tumor was fully removed with clear margins. The tumor was ER/PR positive but Her-2 negative. The right breast histology showed papillomas with florid usual ductal hyperplasia and sclerosis with no residual DCIS. As her final pathology showed a grade 3 left breast tumor, she received adjuvant aromatase inhibitor and 40 Gy EBRT in 15 fractions. The measured absorbed dose resulting from the Zeiss INTRABEAM IORT system radiation on the skin surface was 1.49 (1.32-1.69) Gy for the right breast and 1.19 (1.07-1.34) Gy for the left breast, using the same methodology described previously by our group (24) **Table 2**.

**Table 2:** Dose reported is for the closest skin bridge measurement (applicator to skin distance) as determined using ultrasound measurements localization measuring the 4 cardinal positions of superior, medial, inferior, lateral and has been determined using the validated model presented in Brodin et al. (24) 95% confidence interval is shown in parenthesis.

Patient	Right Breast Closest Skin bridge distance (mm)	Dose to Skin Right Breast (Gy)	Left Breast Closest Skin bridge distance (mm)	Dose to Skin Left Breast (Gy)
Patient 1	15.3	1.49 (1.32-1.69)	17.4	1.19 (1.07-1.34)
Patient 2	14.1	1.73 (1.53-1.97)	12.2	2.27 (2.01-2.59)
Patient 3	16.6	1.29 (1.15-1.46)	16.5	1.30 (1.16-1.47)
Patient 4	7.2	5.48 (4.99-6.03)	9.5	3.57 (3.19-4.01)
Patient 5	14.2	1.71 (1.51-1.95)	14.3	1.68 (1.49-1.92)

### Patient 2

A 72-year-old female, a former smoker, presented with bilateral masses in her breast diagnosed on a screening mammogram. Subsequent ultrasound showed right breast 2:00-3:00 axis 0.5 x 0.4 x 0.4 hypoechoic mass, and the left breast demonstrated a mass at 10:00 axis measuring 1.2 x 1.6 x 1.7. A stereotactic biopsy of the right breast revealed grade 1-2 well-differentiated invasive ductal carcinoma (IDC) with DCIS. The left breast demonstrated IDC with a hyalinized sclerosing lesion. Both the tumors were diagnosed as clinical stage 1 T1 N0 M0 and tumor markers in both breasts were ER +, PR +, and Her-2 negative. Wide local excision was performed on each breast, and two separate IORT treatments were delivered sequentially. Each IORT treatment utilized a 35-mm diameter spherical applicator, delivering a dose of 20 Gy **Table 1**. The measured absorbed dose from the Intrabeam IORT system radiation on the skin surface was 1.73 (1.53-1.97) Gy for the right breast and 2.27 (2.01-2.59) Gy for the left breast **Table 2**.

The surgery and IORT were both uneventful. Histology of the right breast confirmed the presence of IDC measuring 6 mm with DCIS grade 2, stage 1a T1b N0 M0. The left breast was diagnosed with stage 1b T1c N0 M0 grade 1 IDC measuring 17 mm in the greatest dimension. Both the tumors had clear margins on final histology. The patient received an aromatase inhibitor for adjuvant therapy.

### Patient 3

A 73-year-old female with a PMHx of uterine cancer presented following an abnormal mammogram which revealed a 1.7 cm mass at the 3:00 axis and a 7 mm retro-areolar mass in the right breast, as well as calcifications in the left breast at the 4:00 axis. Subsequent ultrasound-guided biopsy demonstrated clinical stage 1a T1, N0, M0 right breast IDC moderately differentiated ER+, PR-, Her-2 negative at 3:00 axis. The retro-areolar mass was diagnosed as fibroadenoma. The left breast biopsy revealed invasive lobular carcinoma (ILC) at 5:00-6:00 axis ER+, PR+, and Her-2 negative with clinical stage 1a T1 N0 M0.

Subsequently, both the breast received IORT treatment after bilateral tumor excision with bilateral SLNB. IORT for the right breast used a 30-mm spherical applicator to deliver 20 Gy over 24 minutes. The left breast IORT treatment used a 35mm applicator to deliver 20 Gy to the surgical margin for 17 minutes **Table 1**. The measured absorbed dose from the Intrabeam IORT system radiation on the skin surface was 1.29 (1.15-1.46) Gy for the right breast and 1.30 (1.16-1.47) Gy for the left breast **Table 2**.

The surgery and IORT were uneventful. Histology of the right breast established a 15 x 8 mm grade 2 IDC with clear margins. The left breast had grade 2 ILC with a maximum size of 16 mm. Both tumors were stage 1a T1 N0 M0 with clear margins on biopsy. Adjuvant treatment with an aromatase inhibitor was initiated.

### Patient 4

A 69-year-old female presented with a 7 mm mass in her left 12:00 axis and microcalcifications in her right outer quadrant on mammogram. The left breast biopsy revealed IDC ER+, PR+, and Her-2 negative, clinical stage 1a T1 N0 M0 and the right breast microcalcifications were clinical stage 0 Tis N0 ER+ DCIS and atypical ductal hyperplasia.

A bilateral breast lumpectomy with left-sided IORT and SLNB was performed. A 35-mm applicator delivered 20 Gy in 17 minutes. Both surgery and IORT were uneventful. The measured absorbed dose from the Intrabeam IORT system radiation on the skin surface was 5.48 (4.99-6.03) Gy for the right breast and 3.57 (3.19-4.01) Gy for the left breast **Table 2**. The pathology of the left breast revealed grade 1 IDC forming an 8 x 7 x 6 mm mass, while the right breast had IDC spanning 3 x 2 mm with grade 2 DCIS. Both tumors were stage 1a T1 N0 M0, ER+PR+, and Her-2 negative and had clear margins. Following the diagnosis of IDC, SLNB and IORT of the right breast was performed. A 30-mm spherical applicator was inserted into the tumor bed and delivered 20 Gy over 24 minutes through the previous incision **Table 1**. The SLNB resulted in right-side negative nodes. Adjuvant treatment with an aromatase inhibitor was initiated.

### Patient 5

A 77-year-old female, a former smoker, presented with a left breast mass, and a subsequent mammogram revealed bilateral masses. Following this, a left breast ultrasound revealed a 2.9 cm mass at 11:00 o’clock and a 1.2 cm right breast mass at 9:00-10:00. Biopsy of the left breast showed poorly differentiated IDC ER+, PR-, and Her-2 negative clinical stage 2 T2 N0 M0 whereas the right breast revealed stage 1 T1 N0 M0 grade 2 invasive carcinoma with mixed ductal and lobular features, ER+, PR+, and Her-2 negative.

Bilateral breast lumpectomy, SLNB, and IORT were all performed. In the right lumpectomy cavity, IORT with a 40-mm spherical applicator delivered 20 Gy for 24 minutes, while in the left cavity, it delivered 20 Gy over 34 minutes **Table 1**. The measured absorbed dose from the Intrabeam IORT system radiation on the skin surface was 1.71 (1.51-1.95) Gy for the right breast and 1.68 (1.49-1.92) Gy for the left breast **Table 2**.

Both the surgery and IORT were uneventful. Pathology demonstrated a grade 3 stage T2 N0 M0 ER+, PR-, Her-2 negative invasive carcinoma with ductal and lobular features spanning 30 mm in the left breast. In contrast, the right breast had grade 2 stage T1 N0 M0 ER+, PR+, Her-2 negative ILC with lobular carcinoma *in situ* spanning 12 mm. Both the tumors had clear margins on final pathology. Adjuvant chemotherapy included TC (Taxotere/Cytoxan) every 3 weeks for 4 cycles and an aromatase inhibitor. At present, she has no evidence of disease on follow-up.

**Figure 1:**
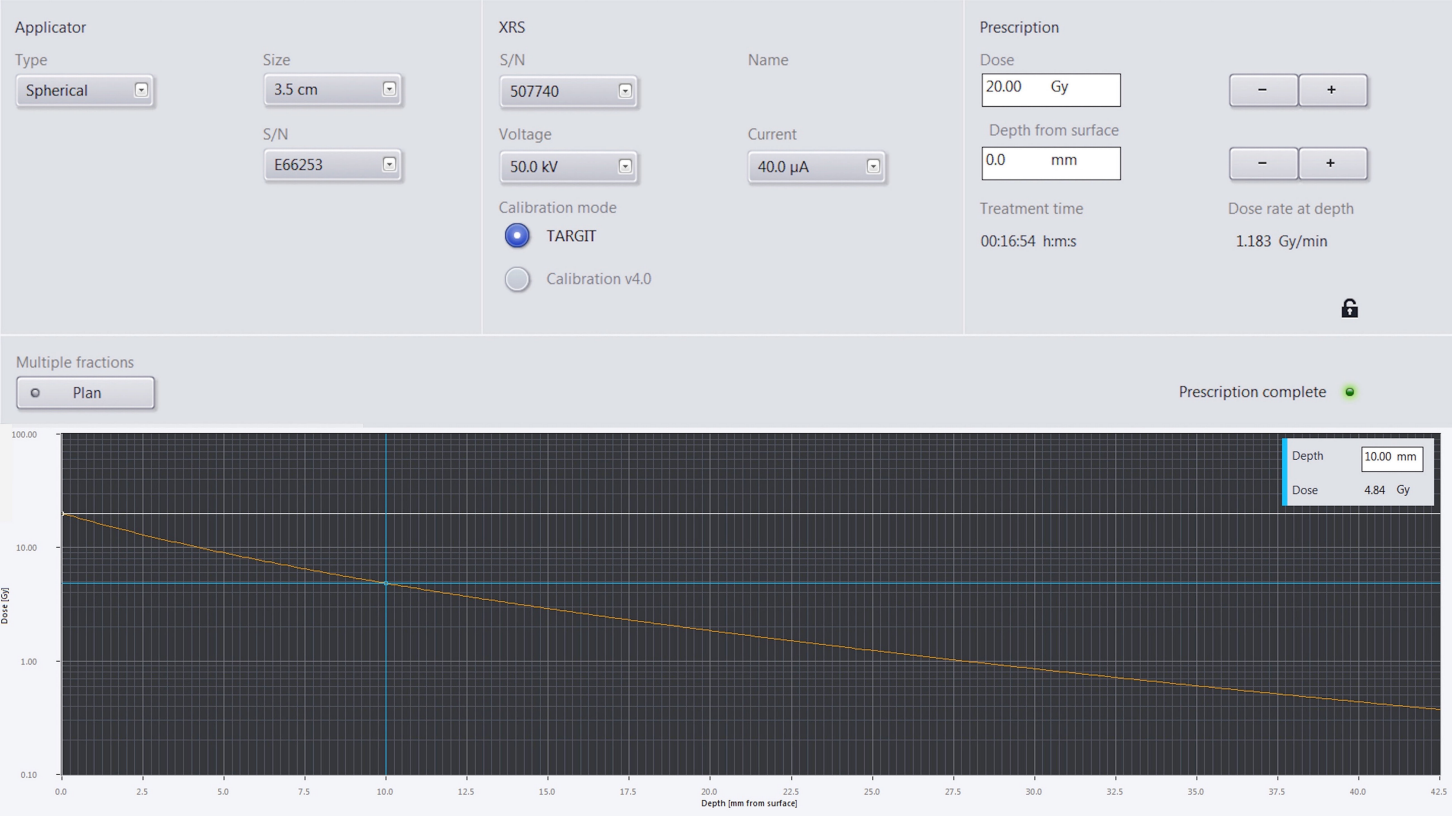
The upper panel of the figure shows the plan parameters used. The left most panel shows the applicator specific information such as applicator size and serial number, the middle panel shows the treatment beam parameters such as beam energy, beam current, and that the calibration mode used, the right most panel shows the prescription dose and at what distance from the applicator surface that dose is prescribed to along with the treatment time and dose rate at depth to deliver the prescribed dose. The bottom panel shows a semi-log plot of the dose versus distance from the applicator surface. The blue cross shows that the dose 10 mm away from the applicator deceases to 4.84 Gy for a prescription dose of 20 Gy at the applicator surface.

Dosimetry demonstrating the prescription and depth dose for one of the applicators is illustrated in **Figure 1**.

## Discussion

IORT is a form of accelerated partial breast irradiation (APBI) that allows for a single high dose of radiation to be delivered directly to the surgical margins shortly after tumor excision. The use of low energy 50kVp photons minimizes scatter and radiation exposure to nearby critical organs due to the steep dose fall-off past the applicator surface; for instance, for a 30 mm applicator, the dose 5mm away from the applicator surface reduces to 49% of the prescriptions dose, while for a distance of 10 mm away from the applicator surfaces it reduces to 28% of the prescription dose (6, 25, 26). This approach to APBI bears similarities to intra-operative electron radiotherapy (IOERT) and balloon-based brachytherapy where IOERT delivers high-energy electrons, with energies reaching up to 12 MeV, to administer radiation treatment (27). Meanwhile, balloon brachytherapy, particularly MammoSite utilizes Ir-192 at a high dose rate to precisely deliver the prescribed radiation dose (28). Traditionally, adjuvant WBI following BCS has been shown to significantly decrease the risk of local recurrence and improve overall survival (29). Furthermore, an additional boost of 10 to 20 Gy to the tumor bed in selected patients has demonstrated a further reduction in the tumor recurrence rate (30, 31). Additionally, there has been a notable shift in the delivery of EBRT with the emergence of hypofractionated radiotherapy, as exemplified in the FAST-FORWARD trial (32). This pivotal randomized controlled phase 3 trial spanned 97 hospitals and included 4,096 patients where they found that implementation of radiotherapy with 26 Gy in five fractions over 1 week is non-inferior to the standard of 40 Gy in 15 fractions over 3 weeks. Despite these advancements, reliable and accurate identification of the tumor bed can be a challenge in the adjuvant setting. It has been shown that the use of the scar and underlying tissue to delineate the boost volume during adjuvant external beam radiation can lead to a partial miss of the CT-defined lumpectomy cavity as determined by surgical clips (33). While, as demonstrated by Coles et al. (34), the use of titanium clips placed in the tumor bed at the time of breast-conserving surgery provides an accurate and reliable method of tumor bed localization, there is still the possibility of inadequately defining the lumpectomy cavity due to post-surgical changes that might lead to either a boost volume that is too small or too large. IORT has the advantage of targeting the lumpectomy cavity intraoperatively, thereby reducing the chance of any inaccuracies in its localization. This is especially relevant for patients undergoing WBI after oncoplastic reconstruction, in whom tissue rearrangement makes accurate localization nearly impossible (35).

The use of WBI has been linked to a number of adverse effects, the most significant of which is non-breast cancer-related mortality (9). WBI has demonstrated an increased risk of secondary cancers and heart disease (36–38). A study of 134 breast cancer patients, 90 of whom underwent WBI, found a rate ratio of 2.10 (95% CI, 1.48 to 2.98; P = 0.001) for lung cancer incidence over ≥ 10 years (36). Moreover, WBI has been linked to a variety of heart diseases, including ischemic heart disease, myocardial infarction, valvular disease, coronary stenosis, pericarditis, and other cardiac abnormalities (36–38). Due to skin toxicity and fibrosis, WBI can worsen cosmetic outcomes, especially when boosting the tumor bed (39). In contrast, IORT significantly reduces non-breast cancer-related mortality rate (45 vs. 74 events for TARGIT-IORT and EBRT, respectively, hazard ratio 0.59; 95% CI, 0.40 to 0.86; P=0.005), including the cardiovascular causes (9). Additionally, in smokers, IORT may reduce the risk of secondary lung cancers that are frequently associated with EBRT. Both Patient 2 and Patient 5 in our study were former smokers but developed no complications during their respective follow-up periods. In terms of toxicities and complications, the TARGIT-A trial randomized 3451 patients to WBI (1730) or IORT (1721). While wound-related complications were similar between groups, IORT had significantly less grade 3 or 4 toxicity and better cosmesis than WBI (22, 40). IORT has also reported better breast-related quality of life and overall quality of life (41, 42). Moreover, IORT can lead to higher patient compliance owing to shorter treatment duration and fewer visits, which may overall lead to a better patient experience (43). Therefore, IORT may be a prudent choice of treatment, particularly in patients with BBC, where there may be a two-fold risk of toxicities and poor cosmesis.

Although previous studies have evaluated the utility of IORT in patients with breast cancer, fewer have elucidated its use in BBC (44–46). Silverstein et al. (44) investigated tumor recurrence and survival rates in 1367 patients who received IORT ± WBI, 33 of whom had BBC. In this study, IORT was delivered using the Xoft Axxent Electronic Brachytherapy System^®^ (Xoft, San Jose, CA, USA, a subsidiary of iCAD, Inc.) following intraoperative balloon placement, a different technique to the Intrabeam system. A total of 60 patients undergoing IORT alone had ipsilateral local recurrence, and their Kaplan-Meier probability of any event over a 5-year follow-up for 1175 patients who received only IORT was 5.98%. A study by Kaiser et al. (46) evaluated the use of intraoperative electron radiation therapy as a boost followed by WBI in breast cancer patients with stages I-III. They included 827 patients in their study over a 10-year period, with 9 patients with BBC. They reported that 21 (2.7%) developed local recurrence, 107 (14%) died, and 106 (14%) developed metastases. While all of these studies included BBC patients as candidates for IORT, none of them specifically highlighted the outcomes of IORT in patients with BBC without WBI. However, our case series illustrates that IORT is a safe and effective treatment option in BBC.

Synchronous BBC irradiation represents a challenge in the current clinical practice due to the large target volume being required for the breast and the contrasting need to minimize the dose to critical organs such as the heart and lungs, and often the esophagus and spinal cord if the supraclavicular nodes require treatment. BBC is a rare disease, and clinical guidelines for BBC irradiation are lacking because, historically, bilateral breast irradiation was only performed in 0.4%-5.5% of patients (17, 47–52). Numerous studies have highlighted the detrimental effects of WBI, but very few have analyzed its outcome in bilateral breast irradiation (53). Rochefordiere et al. (53) reported the outcomes of bilateral WBI in 149 patients who encountered treatment-related complications such as brachial plexopathy, myelitis, various cardiac complications, and rib fractures. However, the majority of the complications can be attributed to the fact that 60% of the patients in their study underwent axillary lymph node dissection. After two years, 48 of 51 patients were assessed for cosmetic outcomes. The study found that 77% (37 patients) had acceptable cosmesis, 15% (7 patients) had fair, and 8% (4 patients) had poor (53). Although recent technological advancements have improved WBI, APBI has been shown to outperform in terms of cosmesis in most, but not all studies (54–59) A study by Rodriguez et al. (54) compared WBI to APBI and observed that with APBI, grade 2 acute dermatitis was reduced from 62.7% to 17.4%, as were radiation doses to vital organs (P <.01). Cosmetic outcomes were reported to be excellent/good in more than 75% and 84% of patients in the APBI and WBI arms, respectively. Similarly, Yadav et al. (59) found that WBI and APBI were associated with 15% and 8% of acute grade ≥2 dermatitis, respectively. Moreover, patients in the APBI arm had a 5% induration rate and a 3% fibrosis rate (59). According to Polgar et al. (55) 2.2% of patients with APBI had grade 3 fibrosis and 7% had grade 2 induration. Another study by the same group found that APBI had 77.6% excellent/good cosmesis and WBI 62.9% (56). In contrast, the RAPID trial (57) found that when compared to standard WBI, APBI resulted in an increase in adverse rates from 17% to 29% (P<.001) over three years, with increased late radiation toxicity. Furthermore, NSABP B-39/RTOG 0413 reported poor clinical outcomes with APBI versus WBI, emphasizing 10-year grade 3 toxicity of 7.1% in the WBI arm and 9.6% in the APBI arm (58).

Despite the lack of consensus, the use of IORT for BBC is a promising option for these patients. TARGIT-IORT as a form of APBI has shown superiority in terms of quality of life and better patient-reported outcomes for cosmesis, breast-related quality of life, and breast pain (41, 42, 60, 61). Keshtegar et al. (60), examined frontal digital breast photos taken before TARGIT-IORT or EBRT and annually for up to 5 years. They calculated a composite score based on symmetry, color, and scar using a software and found that patients in the TARGIT-IORT group had a higher chance of an excellent/good outcome than those in the EBRT group at year 1 (OR 2.07, 95% CI 1.12-3.85, p = 0.021) and year 2 (OR 2.11, 95% CI 1.0-4.45, p = 0.05) (60). Additionally, Andersen et al. (61) conducted a study comparing persistent pain after IORT vs. WBI and observed that 33.9% of patients in the EBRT group reported persistent pain in the breast area, side of the chest, axilla, or arm, compared to 24.6% in the IORT group (P = 0.11). Similarly, the use of IORT in patients with BBC yielded positive results in our study. Elderly patients, such as patients 2, 3, 4, and 5, in our study, may have benefited from surgery and endocrine therapy while avoiding radiation treatment (62). However, the decision to administer radiotherapy to these patients was influenced by the findings of the Cancer and Leukemia Group B (CALGB) 9343 trial, which demonstrated that combining radiation therapy with endocrine therapy resulted in improved locoregional recurrence prevention in women aged ≥ 70 years (63). Furthermore, the PRIME II study conducted a randomized trial involving 1,326 patients with non-metastatic hormone receptor-positive breast cancer who were all at least 65 years old, had BCS, and were receiving adjuvant hormone therapy (21). Their findings revealed a significantly higher rate of local recurrence after 10 years in patients who did not receive radiation therapy compared to those who did (9.8% vs. 0.9%), supporting our decision to include radiotherapy in our patient’s treatment plan. Furthermore, none of the 5 patients experienced any acute or chronic toxicities following BCS and bilateral IORT, including Patient 1, who received EBRT after a pathology grade 3 diagnosis, as per TARGIT-A protocol (9). While ASTRO considers the application of partial breast radiotherapy in ER+ lower-grade *in situ* disease (23), however, to the best of the authors’ knowledge, no studies have been published highlighting the utility on Zeiss Intrabeam system (50 Kv) in DCIS management. Additionally, all of the patients in our study tolerated the IORT well and, after a 36-months median follow-up, developed no local recurrence confirmed via mammography. As the number of breast cancer cases increases, there may be a higher probability of encountering BBC; thus, future studies are required to further evaluate the utility of IORT vs. WBI for BBC in order to establish new guidelines.

Our study has several limitations. First, our study has a small sample size. Second, the study’s short follow-up period limited our ability to capture the long-term effects and outcomes of bilateral IORT. Moreover, the retrospective nature of the study introduces inherent limitations. Finally, our study lacked quality-of-life measurements, a cosmesis scale, and patient-reported outcomes, which could have provided a more comprehensive understanding of the treatment effects.

## Conclusion

Our findings demonstrate the successful use of IORT for BCS in patients with BBC. Furthermore, none of the patients in our study experienced any complications, suggesting the feasibility of the use of IORT in BBC. Considering the benefits of improved patient compliance and a reduced number of multiple visits, IORT may serve as an excellent patient-centered alternative for BBC. Future studies are recommended to reinforce the applicability of IORT in patients with BBC.

## Data availability statement

The raw data supporting the conclusions of this article will be made available by the authors, without undue reservation.

## Ethics statement

Institutional IRB does not require ethical approval for case reports. The studies were conducted in accordance with the local legislation and institutional requirements. The participants provided their written informed consent to participate in this study. Written informed consent was obtained from the individual(s) for the publication of any potentially identifiable images or data included in this article.

## Author contributions

FB: Data curation, Formal Analysis, Investigation, Software, Writing – original draft, Writing – review & editing. MM: Conceptualization, Investigation, Methodology, Supervision, Writing – review & editing. AG: Conceptualization, Methodology, Supervision, Writing – review & editing. JP: Data curation, Investigation, Writing – review & editing. AS: Data curation, Investigation, Methodology, Validation, Writing – review & editing. AB: Data curation, Investigation, Methodology, Software, Writing – review & editing. WT: Conceptualization, Data curation, Investigation, Methodology, Resources, Supervision, Validation, Writing – review & editing. JF: Conceptualization, Data curation, Investigation, Methodology, Writing – review & editing. KM: Data curation, Investigation, Methodology, Writing – review & editing. SF: Conceptualization, Investigation, Project administration, Resources, Supervision, Validation, Visualization, Writing – review & editing.
